# Supplementary material for: A Likelihood Approach for Real-Time Calibration of Stochastic Compartmental Epidemic Models
Source: PLoS Comput Biol. 2017 Jan 17;13(1):e1005257. doi: 10.1371/journal.pcbi.1005257 (PMC5240920; doi:10.1371/journal.pcbi.1005257)
Supplement: S1 File — (TAR.GZ) [file pcbi.1005257.s014.tar.gz › HSPH_Online-SI-Revision/MSS10/n10-mild/n10-mild_NEW2_table0.pdf]

1	newly 3 weeks spec	176.	106.72	{17., 199.}	52.5682	0.489788	newly 3 weeks cumul	530.	437.65	{120., 728.}	39.6811	0.264992	newly 5 weeks spec	135.	53.91	{0., 137.}	67.5333	0.996244	newly 5 weeks cumul	825.	565.65	{135., 999.}	43.7661	0.328095	newly 50 weeks cumul	2160.	701.3	{135., 1313.}	67.9102	0.643186
2	newly 3 weeks spec	316.	269.54	{195., 342.}	19.7468	0.101714	newly 3 weeks cumul	1222.	1089.74	{833., 1343.}	16.0753	0.0795304	newly 5 weeks spec	183.	121.84	{74., 180.}	36.0656	0.215891	newly 5 weeks cumul	1613.	1398.28	{1068., 1705.}	16.3335	0.0818521	newly 55 weeks cumul	2172.	1633.75	{1246., 1955.}	25.5106	0.133868
3	newly 3 weeks spec	434.	277.01	{184., 365.}	36.1728	0.212413	newly 3 weeks cumul	1373.	1127.	{787., 1417.}	20.2709	0.108697	newly 5 weeks spec	309.	122.34	{68., 169.}	60.4078	0.432228	newly 5 weeks cumul	2076.	1439.85	{1034., 1787.}	30.6431	0.170185	newly 58 weeks cumul	2924.	1645.12	{1233., 2004.}	43.7373	0.258766
4	newly 3 weeks spec	417.	230.28	{152., 318.}	44.777	0.276102	newly 3 weeks cumul	1378.	1038.37	{699., 1384.}	25.8962	0.142554	newly 5 weeks spec	196.	83.38	{34., 131.}	57.4592	0.431232	newly 5 weeks cumul	1896.	1261.36	{874., 1657.}	33.5728	0.189842	newly 53 weeks cumul	2527.	1381.16	{1007., 1772.}	45.3439	0.273595
5	newly 3 weeks spec	111.	108.57	{75., 144.}	20.7117	0.0947924	newly 3 weeks cumul	344.	340.85	{252., 418.}	15.1715	0.068105	newly 5 weeks spec	94.	94.55	{60., 133.}	24.0106	0.110153	newly 5 weeks cumul	547.	537.24	{392., 662.}	16.2523	0.0737901	newly 33 weeks cumul	2021.	1197.99	{802., 1569.}	40.7229	0.241322
6	newly 3 weeks spec	331.	302.14	{170., 428.}	25.1178	0.134176	newly 3 weeks cumul	1004.	1001.4	{606., 1380.}	26.5339	0.129901	newly 5 weeks spec	169.	189.98	{96., 278.}	35.1124	0.149229	newly 5 weeks cumul	1390.	1444.85	{895., 1956.}	24.5201	0.113785	newly 59 weeks cumul	2050.	1905.46	{1330., 2422.}	17.6829	0.0844575
7	newly 3 weeks spec	277.	222.86	{134., 333.}	29.9061	0.165702	newly 3 weeks cumul	873.	829.17	{520., 1150.}	25.2818	0.123851	newly 5 weeks spec	186.	117.76	{57., 178.}	38.5054	0.267012	newly 5 weeks cumul	1277.	1108.87	{676., 1544.}	24.3798	0.128887	newly 55 weeks cumul	1892.	1375.86	{863., 1859.}	28.9556	0.167511
8	newly 3 weeks spec	269.	219.99	{131., 311.}	26.4498	0.143543	newly 3 weeks cumul	743.	786.2	{442., 1102.}	25.389	0.110458	newly 5 weeks spec	224.	131.33	{74., 193.}	42.6384	0.269286	newly 5 weeks cumul	1216.	1091.61	{631., 1504.}	21.6077	0.108987	newly 50 weeks cumul	3026.	1434.16	{1019., 1907.}	52.6054	0.340372
9	newly 3 weeks spec	476.	299.28	{196., 396.}	37.2479	0.219821	newly 3 weeks cumul	1520.	1214.27	{864., 1509.}	21.7257	0.116637	newly 5 weeks spec	288.	138.29	{71., 205.}	52.4757	0.35949	newly 5 weeks cumul	2165.	1558.7	{1169., 1952.}	28.2688	0.156155	newly 54 weeks cumul	2749.	1805.28	{1300., 2287.}	34.721	0.197385
10	newly 3 weeks spec	159.	179.53	{93., 273.}	36.3082	0.167915	newly 3 weeks cumul	644.	654.96	{356., 943.}	28.0528	0.140179	newly 5 weeks spec	135.	106.26	{47., 157.}	31.1704	0.241079	newly 5 weeks cumul	960.	902.13	{499., 1317.}	26.251	0.145053	newly 51 weeks cumul	1852.	1181.86	{634., 1592.}	36.4125	0.238126
11	newly 3 weeks spec	170.	77.33	{8., 154.}	55.8882	0.566781	newly 3 weeks cumul	592.	456.12	{115., 743.}	36.598	0.253712	newly 5 weeks spec	147.	22.58	{0., 63.}	85.0476	1.52867	newly 5 weeks cumul	872.	518.9	{115., 832.}	42.8716	0.328451	newly 56 weeks cumul	1526.	553.61	{127., 869.}	63.9889	0.541969
12	newly 3 weeks spec	240.	217.64	{126., 301.}	23.4667	0.128198	newly 3 weeks cumul	1103.	1117.41	{669., 1420.}	20.2094	0.100451	newly 5 weeks spec	100.	68.64	{25., 104.}	38.9	0.25456	newly 5 weeks cumul	1384.	1310.9	{815., 1662.}	18.0925	0.0954387	newly 58 weeks cumul	1560.	1387.29	{884., 1726.}	17.5417	0.0958827
13	newly 3 weeks spec	357.	409.46	{335., 483.}	17.4398	0.0682645	newly 3 weeks cumul	1370.	1418.47	{1220., 1610.}	9.5365	0.0403459	newly 5 weeks spec	224.	255.74	{208., 309.}	17.75	0.0690394	newly 5 weeks cumul	1865.	2001.32	{1727., 2249.}	10.6885	0.0438503	newly 50 weeks cumul	2415.	2645.61	{2334., 2894.}	11.1176	0.0451775
14	newly 3 weeks spec	307.	334.18	{254., 388.}	16.6124	0.069581	newly 3 weeks cumul	1011.	1075.58	{873., 1246.}	12.8981	0.0550097	newly 5 weeks spec	225.	243.87	{185., 305.}	19.3289	0.082747	newly 5 weeks cumul	1501.	1607.57	{1301., 1837.}	13.97	0.0596376	newly 51 weeks cumul	2614.	2518.71	{2030., 2820.}	11.6492	0.0559574
15	newly 3 weeks spec	226.	265.26	{133., 406.}	37.3982	0.152701	newly 3 weeks cumul	696.	818.71	{443., 1176.}	35.9986	0.144079	newly 5 weeks spec	226.	194.24	{109., 270.}	25.6106	0.135003	newly 5 weeks cumul	1140.	1246.37	{693., 1743.}	28.9009	0.124056	newly 53 weeks cumul	3240.	1880.87	{1285., 2430.}	41.9485	0.256981
16	newly 3 weeks spec	319.	290.8	{211., 383.}	19.8056	0.0994952	newly 3 weeks cumul	1150.	1080.06	{770., 1361.}	16.8991	0.0820737	newly 5 weeks spec	224.	153.75	{85., 218.}	34.7902	0.205996	newly 5 weeks cumul	1670.	1453.25	{1091., 1835.}	18.3623	0.0935164	newly 54 weeks cumul	2574.	1752.88	{1274., 2193.}	31.9106	0.178461
17	newly 3 weeks spec	105.	216.92	{166., 267.}	106.59	0.307908	newly 3 weeks cumul	312.	523.29	{414., 628.}	67.7212	0.218963	newly 5 weeks spec	82.	314.78	{250., 378.}	283.878	0.578493	newly 5 weeks cumul	477.	1101.79	{868., 1313.}	130.983	0.357932	newly 51 weeks cumul	3040.	7008.72	{6811., 7182.}	130.55	0.362666
18	newly 3 weeks spec	181.	221.67	{115., 331.}	42.1934	0.169262	newly 3 weeks cumul	580.	694.62	{425., 991.}	34.4724	0.136743	newly 5 weeks spec	194.	162.57	{71., 251.}	32.8608	0.191677	newly 5 weeks cumul	959.	1052.03	{618., 1536.}	30.1366	0.132191	newly 51 weeks cumul	1686.	1584.9	{838., 2115.}	23.032	0.124624
19	newly 3 weeks spec	266.	139.68	{69., 207.}	47.4887	0.311076	newly 3 weeks cumul	957.	703.94	{448., 946.}	28.278	0.159353	newly 5 weeks spec	162.	48.79	{17., 81.}	69.8827	0.621838	newly 5 weeks cumul	1331.	837.95	{532., 1084.}	37.1022	0.218133	newly 54 weeks cumul	1835.	903.33	{554., 1167.}	50.7722	0.323483
20	newly 3 weeks spec	319.	236.5	{127., 337.}	30.5831	0.176771	newly 3 weeks cumul	1048.	949.97	{536., 1343.}	23.6746	0.120398	newly 5 weeks spec	244.	104.82	{46., 165.}	57.041	0.41774	newly 5 weeks cumul	1558.	1217.45	{738., 1732.}	25.8081	0.14393	newly 55 weeks cumul	2688.	1394.42	{910., 1844.}	48.1243	0.30458
21	newly 3 weeks spec	299.	237.32	{159., 328.}	24.6957	0.134293	newly 3 weeks cumul	1149.	1023.56	{723., 1316.}	18.9869	0.0952863	newly 5 weeks spec	209.	103.2	{57., 152.}	50.622	0.34402	newly 5 weeks cumul	1607.	1286.8	{927., 1639.}	21.7859	0.115691	newly 55 weeks cumul	2136.	1446.74	{1058., 1813.}	32.4934	0.181524
22	newly 3 weeks spec	242.	211.02	{111., 294.}	24.405	0.139445	newly 3 weeks cumul	748.	726.42	{422., 1000.}	23.4064	0.11412	newly 5 weeks spec	169.	124.97	{62., 179.}	31.5325	0.200609	newly 5 weeks cumul	1116.	1020.76	{634., 1360.}	21.138	0.110526	newly 51 weeks cumul	2163.	1355.48	{906., 1699.}	37.4267	0.223562
23	newly 3 weeks spec	259.	229.49	{100., 340.}	28.1737	0.158696	newly 3 weeks cumul	963.	903.99	{515., 1244.}	24.4434	0.128059	newly 5 weeks spec	207.	110.55	{48., 169.}	46.8551	0.350396	newly 5 weeks cumul	1381.	1180.41	{680., 1611.}	24.2426	0.138079	newly 56 weeks cumul	1616.	1381.25	{822., 1814.}	21.7915	0.127601
24	newly 3 weeks spec	173.	111.93	{11., 256.}	57.5665	0.499896	newly 3 weeks cumul	546.	473.48	{116., 834.}	44.3223	0.252793	newly 5 weeks spec	105.	53.62	{0., 130.}	67.2952	1.0794	newly 5 weeks cumul	774.	603.8	{153., 1134.}	48.0517	0.300853	newly 52 weeks cumul	1235.	730.64	{167., 1466.}	51.0737	0.387359
25	newly 3 weeks spec	260.	747.73	{447., 982.}	187.927	0.436749	newly 3 weeks cumul	915.	2130.74	{1294., 2534.}	134.326	0.355748	newly 5 weeks spec	241.	571.04	{169., 908.}	151.485	0.366495	newly 5 weeks cumul	1474.	3381.98	{2021., 4326.}	130.366	0.342088	newly 59 weeks cumul	2031.	5326.27	{2354., 7666.}	162.489	0.364763
26	newly 3 weeks spec	226.	153.64	{78., 221.}	34.6814	0.245845	newly 3 weeks cumul	712.	621.04	{346., 851.}	25.2163	0.150496	newly 5 weeks spec	102.	76.04	{29., 124.}	39.4314	0.27953	newly 5 weeks cumul	971.	810.47	{465., 1082.}	24.5304	0.152634	newly 50 weeks cumul	1344.	962.15	{575., 1314.}	30.3683	0.197585
27	newly 3 weeks spec	316.	194.5	{112., 269.}	39.2152	0.246135	newly 3 weeks cumul	1146.	912.47	{569., 1201.}	24.5803	0.139332	newly 5 weeks spec	192.	80.94	{24., 137.}	57.8438	0.461994	newly 5 weeks cumul	1600.	1121.72	{680., 1481.}	30.9188	0.180323	newly 52 weeks cumul	1907.	1236.91	{760., 1606.}	35.2748	0.209962
28	newly 3 weeks spec	99.	73.64	{27., 121.}	35.9394	0.231736	newly 3 weeks cumul	324.	261.7	{118., 371.}	27.7469	0.163536	newly 5 weeks spec	91.	54.61	{15., 100.}	47.3956	0.395066	newly 5 weeks cumul	522.	380.34	{174., 545.}	31.4636	0.198876	newly 30 weeks cumul	1626.	620.41	{244., 1081.}	61.8444	0.488135
29	newly 3 weeks spec	301.	217.03	{143., 295.}	29.0465	0.167346	newly 3 weeks cumul	953.	876.57	{627., 1119.}	18.5026	0.0937301	newly 5 weeks spec	234.	102.81	{50., 162.}	56.0641	0.415882	newly 5 weeks cumul	1506.	1133.09	{825., 1441.}	25.7457	0.142124	newly 53 weeks cumul	2280.	1325.8	{946., 1745.}	41.8509	0.250873
30	newly 3 weeks spec	342.	174.45	{100., 251.}	48.9912	0.321182	newly 3 weeks cumul	1048.	709.52	{475., 926.}	32.6183	0.186212	newly 5 weeks spec	206.	86.93	{35., 147.}	57.8398	0.443073	newly 5 weeks cumul	1528.	924.2	{595., 1232.}	39.5223	0.236327	newly 52 weeks cumul	2105.	1101.84	{670., 1563.}	47.6561	0.303082
31	newly 3 weeks spec	270.	425.73	{305., 543.}	60.6852	0.19915	newly 3 weeks cumul	910.	1357.27	{868., 1704.}	52.1418	0.17595	newly 5 weeks spec	207.	259.59	{175., 348.}	31.0966	0.113651	newly 5 weeks cumul	1305.	1962.33	{1460., 2415.}	52.0958	0.175798	newly 59 weeks cumul	1766.	2539.52	{1920., 2973.}	43.8437	0.152869
32	newly 3 weeks spec	121.	120.26	{80., 155.}	18.9091	0.092832	newly 3 weeks cumul	377.	401.93	{295., 490.}	17.435	0.0776803	newly 5 weeks spec	116.	90.37	{43., 131.}	27.5948	0.172634	newly 5 weeks cumul	611.	596.39	{400., 737.}	16.4304	0.081009	newly 32 weeks cumul	2466.	1102.61	{521., 1558.}	55.2875	0.383576
33	newly 3 weeks spec	364.	310.89	{213., 390.}	21.3489	0.106645	newly 3 weeks cumul	1353.	1215.06	{947., 1465.}	15.4959	0.0741793	newly 5 weeks spec	266.	152.6	{82., 211.}	46.6692	0.292019	newly 5 weeks cumul	1933.	1588.51	{1221., 1896.}	21.4646	0.10735	newly 53 weeks cumul	2442.	1889.54	{1385., 2229.}	28.0663	0.142311
34	newly 3 weeks spec	262.	231.34	{118., 350.}	29.0076	0.16598	newly 3 weeks cumul	877.	891.99	{456., 1308.}	28.0331	0.140153	newly 5 weeks spec	247.	117.74	{61., 177.}	52.332	0.36309	newly 5 weeks cumul	1378.	1176.6	{689., 1723.}	24.7939	0.140998	newly 53 weeks cumul	2645.	1406.66	{860., 1924.}	46.8181	0.299316
35	newly 3 weeks spec	285.	188.59	{105., 260.}	34.2281	0.203438	newly 3 weeks cumul	970.	820.76	{587., 1026.}	19.4969	0.103007	newly 5 weeks spec	184.	81.41	{30., 130.}	56.0054	0.41387	newly 5 weeks cumul	1379.	1029.56	{702., 1315.}	25.868	0.142975	newly 53 weeks cumul	2032.	1178.07	{798., 1539.}	42.0241	0.250632
36	newly 3 weeks spec	365.	192.56	{110., 266.}	47.2438	0.30073	newly 3 weeks cumul	1132.	922.04	{589., 1220.}	22.4611	0.120829	newly 5 weeks spec	206.	70.84	{28., 117.}	65.6117	0.521461	newly 5 weeks cumul	1604.	1109.01	{760., 1423.}	30.9757	0.173516	newly 55 weeks cumul	2018.	1214.76	{863., 1531.}	39.8038	0.231773
37	newly 3 weeks spec	233.	325.95	{188., 438.}	46.588	0.162835	newly 3 weeks cumul	842.	1282.91	{749., 1715.}	58.6568	0.192941	newly 5 weeks spec	124.	141.18	{74., 219.}	34.1935	0.137294	newly 5 weeks cumul	1099.	1649.28	{1060., 2106.}	54.2202	0.181274	newly 58 weeks cumul	1455.	1869.08	{1291., 2314.}	33.8034	0.125792
38	newly 3 weeks spec	329.	230.56	{151., 312.}	30.541	0.172778	newly 3 weeks cumul	996.	886.37	{585., 1180.}	20.3725	0.101826	newly 5 weeks spec	222.	122.05	{62., 183.}	45.0225	0.292337	newly 5 weeks cumul	1520.	1178.77	{817., 1543.}	24.1783	0.129879	newly 54 weeks cumul	2847.	1422.32	{996., 1888.}	50.0414	0.31287
39	newly 3 weeks spec	207.	215.31	{129., 293.}	25.8116	0.115143	newly 3 weeks cumul	1062.	997.32	{573., 1359.}	23.4576	0.120377	newly 5 weeks spec	83.	74.71	{32., 124.}	34.6145	0.183027	newly 5 weeks cumul	1296.	1199.38	{738., 1592.}	21.2901	0.110127	newly 55 weeks cumul	1395.	1297.86	{769., 1699.}	20.7599	0.107035
40	newly 3 weeks spec	80.	90.27	{56., 129.}	29.8625	0.122207	newly 3 weeks cumul	250.	291.47	{213., 369.}	24.42	0.0959052	newly 5 weeks spec	81.	74.72	{40., 118.}	31.4568	0.1594	newly 5 weeks cumul	408.	447.95	{289., 602.}	23.326	0.0966893	newly 28 weeks cumul	1434.	928.77	{517., 1326.}	36.5502	0.22659
41	newly 3 weeks spec	318.	288.98	{183., 387.}	22.2138	0.113014	newly 3 weeks cumul	1244.	1265.59	{790., 1709.}	23.1278	0.105616	newly 5 weeks spec	73.	111.2	{60., 161.}	60.5205	0.199311	newly 5 weeks cumul	1452.	1558.48	{1060., 2058.}	22.5992	0.0978728	newly 59 weeks cumul	1481.	1704.81	{1212., 2139.}	24.5192	0.0993037
42	newly 3 weeks spec	264.	237.4	{143., 334.}	25.6136	0.126771	newly 3 weeks cumul	803.	827.37	{544., 1128.}	22.2154	0.0972506	newly 5 weeks spec	207.	134.65	{62., 193.}	35.9469	0.22513	newly 5 weeks cumul	1233.	1145.47	{759., 1535.}	20.7145	0.0996782	newly 51 weeks cumul	1934.	1460.42	{954., 1983.}	26.3278	0.144866
43	newly 3 weeks spec	267.	228.64	{118., 322.}	24.5618	0.135419	newly 3 weeks cumul	1014.	1067.28	{663., 1431.}	25.2525	0.113928	newly 5 weeks spec	120.	87.27	{44., 135.}	33.9417	0.206438	newly 5 weeks cumul	1409.	1296.74	{879., 1698.}	19.6749	0.100939	newly 57 weeks cumul	1476.	1411.71	{1007., 1760.}	17.395	0.0862097
44	newly 3 weeks spec	266.	226.39	{130., 311.}	24.297	0.134939	newly 3 weeks cumul	845.	797.43	{458., 1060.}	21.1444	0.107919	newly 5 weeks spec	232.	131.57	{69., 193.}	44.4784	0.284783	newly 5 weeks cumul	1322.	1105.7	{780., 1456.}	21.3419	0.117587	newly 53 weeks cumul	2625.	1435.12	{987., 1876.}	45.3288	0.278543
45	newly 3 weeks spec	490.	620.72	{365., 741.}	34.7592	0.134378	newly 3 weeks cumul	1749.	2303.77	{1797., 2682.}	34.0818	0.124891	newly 5 weeks spec	208.	266.11	{118., 366.}	41.2452	0.161939	newly 5 weeks cumul	2301.	2993.47	{2188., 3453.}	33.9874	0.126763	newly 60 weeks cumul	2545.	3367.79	{2272., 3856.}	36.9914	0.137479
46	newly 3 weeks spec	320.	312.75	{185., 417.}	22.0969	0.110812	newly 3 weeks cumul	1046.	1096.16	{709., 1424.}	23.3021	0.107095	newly 5 weeks spec	217.	172.24	{96., 247.}	27.8249	0.167168	newly 5 weeks cumul	1558.	1508.05	{1010., 1900.}	18.7478	0.0949235	newly 57 weeks cumul	1999.	1881.33	{1294., 2400.}	16.6808	0.0886951
47	newly 3 weeks spec	449.	503.99	{287., 791.}	32.7595	0.130401	newly 3 weeks cumul	1325.	1605.14	{962., 2219.}	33.1079	0.128159	newly 5 weeks spec	304.	322.42	{127., 695.}	57.0461	0.242047	newly 5 weeks cumul	2024.	2346.75	{1450., 3696.}	32.3542	0.124753	newly 57 weeks cumul	2952.	3244.82	{1841., 6215.}	44.3137	0.172961
48	newly 3 weeks spec	220.	277.95	{146., 383.}	44.4955	0.179291	newly 3 weeks cumul	864.	1058.68	{513., 1507.}	38.5718	0.156089	newly 5 weeks spec	200.	124.99	{60., 192.}	39.615	0.277045	newly 5 weeks cumul	1267.	1368.25	{698., 1861.}	27.8571	0.126095	newly 54 weeks cumul	1825.	1576.19	{921., 2043.}	20.2603	0.1127
49	newly 3 weeks spec	192.	203.76	{76., 331.}	41.5938	0.195473	newly 3 weeks cumul	594.	692.07	{307., 1115.}	40.5572	0.164948	newly 5 weeks spec	164.	134.34	{44., 218.}	35.378	0.316267	newly 5 weeks cumul	949.	994.46	{388., 1553.}	34.6301	0.158661	newly 50 weeks cumul	2640.	1409.04	{555., 2036.}	46.6273	0.315654
50	newly 3 weeks spec	145.	147.2	{76., 228.}	31.5448	0.146345	newly 3 weeks cumul	506.	567.14	{321., 786.}	30.6719	0.12912	newly 5 weeks spec	93.	79.11	{31., 141.}	40.3333	0.248596	newly 5 weeks cumul	715.	755.41	{449., 1045.}	27.4336	0.121666	newly 50 weeks cumul	1140.	939.51	{582., 1257.}	24.8904	0.137365
